# The United States Food and Drug Administration (FDA) regulatory response to combat neglected tropical diseases (NTDs): A review

**DOI:** 10.1371/journal.pntd.0011010

**Published:** 2023-01-12

**Authors:** Sanjana Mukherjee

**Affiliations:** Center for Global Health Science and Security, Department of Microbiology and Immunology, Georgetown University, Washington, District of Columbia, United States of America; Golestan University of Medical Sciences and Health Services, ISLAMIC REPUBLIC OF IRAN

## Abstract

The availability and accessibility of safe and effective drugs, vaccines, and diagnostics are essential to reducing the immense global burden of neglected tropical diseases (NTDs). National regulatory authorities, such as the United States Food and Drug Administration (FDA), play an essential role in this effort to ensure access to safe and effective medical products by working within a set of legal frameworks and regulatory functions. However, medical product development for NTDs remains neglected, as combating NTDs is not a viable commercial market for pharmaceutical companies. To spur research and development (R&D) of NTD products, the US government has authorized various programs and policies to engage pharmaceutical companies, many of which provide FDA with the legal authority to implement NTD programs and pathways. Thus, this review provides a clear overview of the various regulatory pathways and programs employed by the FDA to increase the availability of NTD drugs, vaccines, and diagnostics. The review assesses the available information on various regulatory considerations and their impact on NTD product development as a first step in estimating the importance of such programs. Next, findings related to currently approved NTD products through these programs are discussed. Lastly, gaps in NTD R&D are identified and suggestions on how to address these are presented. The available data shows that while such incentive programs are factored into companies’ decisions to pursue NTD R&D, approved products for NTDs remains vastly insufficient. Most approved products that utilize these NTD regulatory pathways and programs are overwhelmingly for tuberculosis and malaria—both of which are not considered NTDs by the World Health Organization (WHO). Dedicated efforts are needed to facilitate and accelerate NTD product including employing multiple incentive programs, regular assessment of such programs, and leveraging on public–private partnerships.

## Introduction

Neglected tropical diseases (NTDs) are a group of diseases that have an immense global health burden, with one-sixth of the world’s population impacted by one or more NTDs [1]. Infection with an NTD may result in chronic effects such as severe disability, disfigurement, and malnutrition; indeed, the disability-adjusted life year (DALY) for NTDs was estimated at 26.06 million DALYs [2]. Additionally, NTDs have severe economic impacts resulting from damaging effects on the ability to work, on overall child development, on attendance in schools and learning, and on cost of treatment [3]. Currently, 20 diseases and/or conditions are recognized as NTDs by the World Health Organization (WHO); the latest additions to this list are chromoblastomycosis and other deep mycoses, scabies, and other ectoparasites and snakebite envenoming, added in 2017 [4]. While NTDs affect some of the world’s poorest and marginalized communities living predominantly, but not exclusively, in tropical and subtropical countries in Africa, Asia, and the Americas [5], they have also been observed, and can have a substantial public health impact, in countries where they are generally not considered endemic. For instance, the United States Centers for Disease Control and Prevention (CDC) estimates that approximately 300,000 people in the US are affected by Chagas disease, an NTD caused by the parasite *Trypanosoma cruzi* [6].

Access to quality assured medical products such as drugs, vaccines, and diagnostics is key for the treatment, control, and prevention of NTDs. Despite the critical role of such medical products in tackling NTDs, there is a significant gap in the availability of such products. To ensure the availability of such life-saving medical products, a global response to NTDs is required. This review gives an account of how the global response to NTDs was built, the need for placing NTD medical products research and development (R&D) on the global health agenda, and the role of the US government in spurring R&D of NTD products through its national regulatory authority, the US Food and Drug Administration (FDA).

### Global policy response to tackle NTDs

In September 2000, 8 Millennium Development Goals (MDG) were adopted by the United Nations and its member states; the goal of MDG 6 was to specifically “Combat HIV/AIDS, Malaria, and other major diseases” [7]. However, MDG 6 led to concentrated efforts on the “big three”—HIV/AIDS, malaria, and tuberculosis, and sidelined “other diseases” that have debilitating effects, especially among the extreme poor [1,8–10]. This prompted coordination of specific efforts to direct resources to address NTDs. The notion of grouping NTDs and integrating control efforts was conceptualized in 2003 at a WHO/Deutsche Gesellschaft für Technische Zusammenarbeit (GTZ) meeting [8,11,12]. In 2003, the Foundation for Innovative Diagnostics (FIND) was created with the goal of “accelerating the development, evaluation, and delivery of high-quality, affordable diagnostic tests for poverty-related diseases” [13]. Drugs for Neglected Diseases initiative (DNDi) was also founded in 2003 to “discover, develop, and deliver new treatments for neglected patients around the world that are affordable and patient-friendly” by bringing together public, private, academic, nonprofit, and philanthropic sectors [14]. In 2005, the WHO created a department for NTDs and, in 2006, the Global Network for Neglected Tropical Diseases (GNNTDC) was created to raise awareness, political will, and funding to combat the most common NTDs. Additionally, countries responded to this immense challenge by developing policies and forming national objectives to fight NTDs, while international stakeholders pledged support by signing the London Declaration on January 30, 2012 [15]. The signing of the London Declaration on Neglected Tropical Diseases by governments, nongovernmental organizations, pharmaceutical companies, and other stakeholders was a pivotal moment in the fight against NTDs. The Declaration defined multiple key goals including spurring R&D of NTD treatments and tools, enhancing collaboration and coordination on NTDs at national and international levels, providing technical support to countries with significant NTD burdens, and ensuring sustainability of NTD control programs [16].

### US government efforts to combat NTDs

Over the past 20 years, global health has become a significant priority in US foreign policy, with US Congress supporting funding for many global health programs worldwide. NTDs are an important focus of US’s global health efforts, with programs and funding for NTDs increasing significantly since the early 2000s. In 2006, the United States Agency for International Development (USAID) launched its Neglected Tropical Disease (NTD) Control Program in response to the FY2006 Foreign Operations Appropriations Act “to support an integrated response to the control of neglected diseases including intestinal parasites, schistosomiasis, lymphatic filariasis, onchocerciasis, trachoma, and leprosy” [17]. The NTD Program was launched in countries with significant NTD burden to assist national governments develop and scale-up cost-efficient NTD programs that integrate preventive chemotherapy into national initiatives, thus creating a sustainable and long-term impact on NTD control and elimination [18,19]. Total US funding for the USAID NTD Program increased significantly from $15 million in 2006 to $102.5 million in 2020 [20]. In addition to USAID, CDC is also involved in efforts to reduce the burden of 7 NTDs by supporting mass drug administration (MDA) efforts, developing global policies and guidelines for NTD control, and providing technical assistance to partner countries [21,22]. The National Institutes of Health (NIH) has actively been involved in research efforts to develop new therapeutics for control of NTDs [23]. In 2009, NIH launched a $24 million program called the Therapeutics for Rare and Neglected Diseases (TRND) program that “stimulates therapeutic development research collaborations among NIH and academic scientists, nonprofit organizations, and pharmaceutical and biotechnology companies working on rare and neglected illnesses” [24]. The Department of Defense (DOD) is another key agency involved in NTD eradication efforts by funding the development of vaccines for NTDs and evaluating techniques and tools for NTD control [25].

### Efforts (and lack thereof) to stimulate R&D for NTD products

In November 2020, the 73rd World Health Assembly endorsed the document “Ending the neglect to attain the Sustainable Development Goals: a road map for neglected tropical diseases 2021−2030” that set global targets and milestones to fight NTDs. This 2030 NTD Roadmap recognizes that access to safe, effective, and quality-assured medical products including diagnostics, drugs, and vaccines are an essential component of the response against NTDs and requires coordinated and sustained action [26]. Although medical products remain an essential component of the NTD toolkit, studies have shown that R&D programs for NTD medical products remain vastly insufficient [27,28]. The R&D process of bringing medical products to the market is time consuming and expensive, with many potential candidates never making it to market. Drug and vaccine candidates must go through various steps of R&D from laboratory development to preclinical research to clinical trials and through regulatory approval—this process can take at least 10 years with an average cost of $2.6 billion [29]. To determine whether to begin the R&D process of a new product, pharmaceutical companies assess the anticipated lifetime global revenue of a therapeutic, anticipated costs to develop a new product and policies or programs that may impact the supply and demand of the product [30]. Since NTDs mostly affect people living in lower income countries, developing products to treat these diseases is deemed unprofitable by pharmaceutical companies. While cases of NTDs are also observed in high-income countries, the rare occurrence of such cases disincentives companies due to limited market volumes. Additionally, the lack of commercial incentives offered discourages pharmaceutical companies from investing in NTD product development. In keeping with the goal of advancing R&D for NTDs, governments and foundations, worldwide, have developed incentives for NTD therapeutic development [31–33]. These incentives fall under 2 categories. First, “pull mechanisms” provide market sustainability or rewards for successful development and approval of the product. This could include advance market commitments (AMCs), priority review vouchers, and market exclusivity or patent extension rewards. Second, “push mechanisms” provide up-front support and funding to pharmaceutical companies to reduce costs associated with R&D and foster innovation. This could include tax credits, direct funding or grants, and access to intellectual property.

Regulatory authorities, such as FDA, play a critical role in working with companies during the early R&D stages and the product development stage to develop lifesaving medical products and addressing critical gaps in product development and availability. FDA also collaborates with national and global stakeholders either through involvement in review groups or working with procurement agencies to ensure access to drugs, vaccines, and devices. While previous literature has examined the role of the US government in tackling NTDs through agencies such as USAID, CDC, NIH, or DOD, a review of regulatory efforts and policies to ensure access to safe and effective medical products to combat NTDs is warranted. A review of such efforts undertaken by national regulatory authorities, like the FDA, can better inform future regulatory frameworks to improve access to lifesaving NTD medical products. Thus, this review provides a comprehensive and up to date description of the US government’s regulatory response to tackle NTDs through the FDA, identifies successes and gaps in such regulatory frameworks, and provides suggestions on improving access to lifesaving medical products.

## Methods

Information for this review was extracted from: (1) academic publications and reports using PubMed and Google Scholar; (2) websites, reports, and publicly available databases from the US government, including the US FDA, Federal Register, US Government Accountability Office, US Securities and Exchange Commission, and Office of Inspector General; (3) websites and reports from think tanks; (4) websites and reports from private pharmaceutical companies, or public institutions producing NTD products; and (5) local and international news media reports. The search was conducted between June and July 2022 and was limited neither by language, study design, type of publication, nor date of publication.

## Results

### US FDA regulatory considerations to combat NTDs

**Fig 1:**
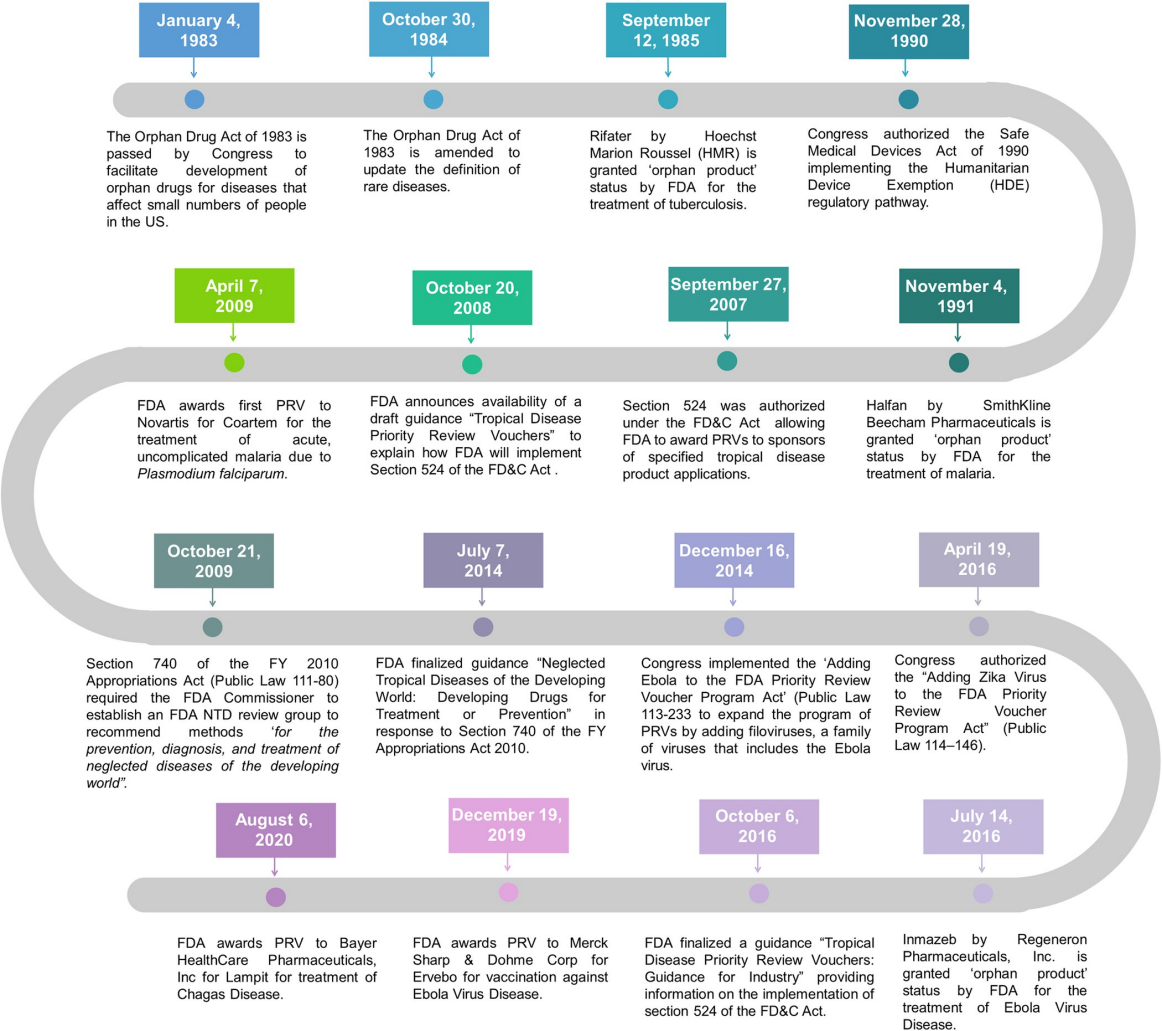
Key milestones in the US FDA’s role to combat NTDs. Information to create this figure was adapted from FDA websites, FDA databases and approval packages and the Library of Congress Public Laws. FDA, Food and Drug Administration; FD&C, Federal Food, Drug, and Cosmetic; HDE, Humanitarian Device Exemption; NTD, neglected tropical disease; PRV, priority review voucher.

The US FDA, a federal agency of the Department of Health and Human Services, is authorized by the United States Federal Food, Drug, and Cosmetic (FD&C) Act to regulate and oversee the safety of human and veterinary drugs, biological products, medical devices, food, and cosmetics. Apart from reviewing applications for drugs, biological products, and devices to ensure access to safe and effective NTD products, FDA operates multiple programs, authorized by Congress, to incentivize pharmaceutical companies to develop NTD products. Furthermore, FDA also publishes guidance documents providing companies and other stakeholders with its interpretation of policies on various regulatory issues ranging from manufacturing to labeling to applying for incentive programs. The current regulatory mechanisms, policies, and activities are listed below and a timeline of key regulatory events to address NTDs through FDA is presented in Fig 1.

### The tropical disease Priority Review Voucher (PRV) program

In 2006, Ridley and colleagues proposed the development of “priority review vouchers” as incentives to drug companies to develop treatment options for neglected diseases [34]. Since most people suffering from NTDs live in low-income countries, the authors argued that financial incentives for drug companies to develop therapies and enter costly clinical R&D for such diseases were lacking. To address this gap in the development of therapeutics for NTDs, in 2007, the US Congress added Section 524 “Priority review to encourage treatments for tropical diseases” to the Federal Food, Drug, and Cosmetic Act [21 USC §360n] to enhance the number of approvals for products in the US [35,36]. A full list of diseases that qualify as “tropical diseases” under the FD&C Act is available in Table 1; 8 diseases, including tuberculosis and malaria, are considered “tropical diseases” by the FD&C Act but are not defined as NTDs by WHO. This voucher is issued by the US FDA to a company when its tropical disease PRV-eligible drug or biological product has been approved. The holder of this voucher can designate a single human drug or biological product application for priority review. If an application is designated as “priority review,” FDA reviews applications within 6 months, compared to 10 months under “standard review.” FDA enables these “priority review” applications by directing attention and resources to the evaluation of these applications, paving the way for quicker patient access if approved.

**Table 1:** List of diseases or conditions recognized by the WHO and US FDA as NTDs and tropical diseases, respectively.

Disease or condition	Recognized as NTD by WHO	Recognized as tropical diseases by FDA^£^
Brucellosis	No	Yes
Buruli ulcer	Yes	Yes
Chagas disease	Yes	Yes
Cholera	No	Yes
Cryptococcal meningitis	No	Yes
Dengue and Chikungunya	Yes	Yes
Dracunculiasis (Guinea-worm disease)	Yes	Yes
Echinococcosis	Yes	No
Filovirus diseases	No	Yes
Foodborne trematodiases	Yes	Yes (only fascioliasis, opisthorchiasis, and paragonimiasis)
Human African trypanosomiasis (sleeping sickness)	Yes	Yes
Lassa fever	No	Yes
Leishmaniasis	Yes	Yes
Leprosy (Hansen’s disease)	Yes	Yes
Lymphatic filariasis	Yes	Yes
Malaria	No	Yes
Mycetoma, chromoblastomycosis, and other deep mycoses	Yes	No
Onchocerciasis (river blindness)	Yes	Yes
Rabies	Yes	Yes
Scabies and other ectoparasitoses	Yes	No
Schistosomiasis	Yes	Yes
Soil-transmitted helminthiases	Yes	Yes
Snakebite envenoming	Yes	No
Taeniasis/cysticercosis	Yes	Yes (only neurocysticercosis)
Trachoma	Yes	Yes
Tuberculosis	No	Yes
Yaws and other endemic treponematoses	Yes	Yes (only Yaws)
Zika virus disease	No	Yes

^£^ Current list of diseases and conditions recognized by FDA as “tropical diseases” present in Section 524(a)(3) of the FD&C Act.

FDA, Food and Drug Administration; FD&C, Federal Food, Drug, and Cosmetic; NTD, neglected tropical disease; WHO, World Health Organization.

To qualify for a PRV, a company’s application must be for a “tropical disease” drug or biological product, must itself qualify for a priority review, and must not contain any active ingredient that has been approved in any other application under Section 505(b)(1) of the FD&C Act (regulatory pathway for new drug applications, NDA) or Section 351 of the Public Health Services Act (the regulatory pathway for biologics license applications, BLA) [37]. Upon submission of an eligible drug or biologics application to FDA and a request for a voucher by the pharmaceutical company, FDA determines whether to approve the drug or biological product application and the voucher request. Once FDA awards the PRV to the company with the approved tropical disease product, the company can either redeem the voucher for priority review a future drug or biological product application, or the company can sell or transfer the PRV to another pharmaceutical company [38]. PRVs may be sold or transferred any number of times before they are redeemed [36]. However, companies redeeming PRVs must pay a PRV user fee in addition to any other fee required under the Prescription Drug User Fee Act (PDUFA) to help FDA incur costs related to priority reviews. To assist pharmaceutical companies on tropical disease PRVs, FDA published the “Tropical Disease Priority Review Vouchers: Guidance for Industry” guidance on October 6, 2016 [36].

**Fig 2:**
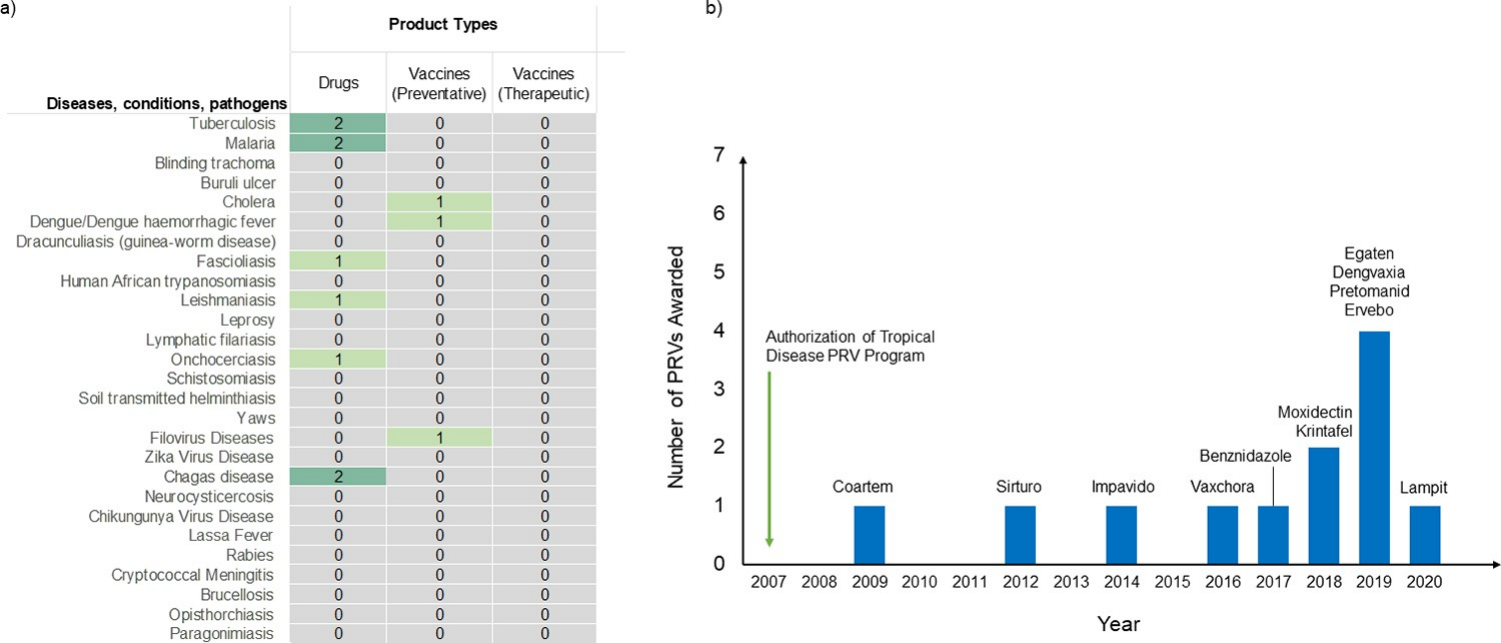
Products approved for the tropical disease PRV by the US FDA. (a) Diseases eligible for the tropical disease PRV program and number of products approved for PRV by FDA, by disease and product type. (b) Products approved, by year, since implementation of the program. Data to generate figure was extracted from FDA approval letters (Drugs@FDA), US SEC filings (EDGAR database using Form 6-K, Form 8-K, and Form 10-K), and US GAO reports. FDA, Food and Drug Administration; GAO, Government Accountability Office; PRV, priority review voucher; SEC, Securities and Exchange Commission.

Since the start of the PRV program in 2007, only 12 products have been approved and issued tropical disease PRVs as of 2020 (Fig 2). Of these products, 9 are drugs while 3 are vaccines, with highest number of approvals and PRV issuance for products against tuberculosis, malaria, and Chagas disease; of these 3 diseases, tuberculosis and malaria are not considered NTDs by WHO. The first PRV was issued in 2009 to Novartis for Coartem (S1 Table), a now widely used antimalarial medicine. While some companies, who were awarded a PRV, redeemed the vouchers for their own products of choice, other companies sold the issued PRV to another company. For instance, Novartis used its PRV (issued for Coartem) to apply for a priority review of its product Ilaris [39]. Janssen Biotech also redeemed its PRV (issued for Sirturo) to speed up the review of Tremfya, which was subsequently approved by the FDA for the treatment of moderate-to-severe plaque psoriasis [39,40]. On the other hand, Knight Therapeutics, a Paladin Labs spin-off, sold its PRV (issued for Impavido) to Gilead Sciences for $125 million [41]. Recently, in 2020, the biotechnology company Argenx SE entered into an asset purchase agreement with Bayer Healthcare Pharmaceuticals to purchase the PRV issued for Lampit [42].

The devastating 2014 to 2016 Ebola outbreak in West Africa further highlighted the need for directing resources for the development of lifesaving vaccines and drugs to combat emerging infectious diseases. To address the lack of therapeutic products targeting such emerging infectious diseases, President Barack Obama signed into law the “Adding Ebola to the FDA Priority Review Voucher Program Act” on December 16, 2014 [43]. Five years later, on December 19, 2019, the Ebola Zaire vaccine Ervebo, manufactured by Merck, was approved by FDA, and awarded a tropical disease PRV. Zika virus also spread rapidly in 2015 to 2016, causing significant morbidity especially in Latin America and the Caribbean; on April 19, 2016, the “Adding Zika Virus to the FDA Priority Review Voucher Program Act” was also signed into law [44]. Apart from adding additional diseases to Section 524(a)(3) of the FD&C Act by legislation, FDA also adds diseases by order under 524(a)(3)(S) (37). Recently, in 2020, FDA added 2 foodborne trematode infections, opisthorchiasis and paragonimiasis, to the list of designated tropical diseases whose product applications may result in the issuance of PRVs.

Several studies have examined the impact of the issuance of PRVs as an incentive to spur R&D of NTD drugs and vaccines. While companies involved in the development of NTD PRV eligible products indicated that the issuance of PRVs was a major or strong consideration of the companies’ decision to continue an NTD development project, “potential market value in the developing world or emerging markets” remained the most important factor when starting or continuing an NTD project [45]. Additionally, Kerr and colleagues reported a significant increase in the development of tropical disease drugs since the authorization of the program in 2007, with 523 preclinical and clinical development programs commencing as of 2016 [46]. However, using a difference-in-difference-in-differences regression analysis, Aerts and colleagues did not observe a statistically significant association between the authorization of the program and the beginning of tropical disease clinical trials extracted from the WHO International Clinical Trials Registry Platform [39]. Although the impact of PRVs on NTD product development may not be clear, experts agree that while the tropical disease PRV program has its merits, it should be refined and/or supplemented with additional incentives to stimulate innovation [33,39,47,48]. A major concern of the program is that, although the program offers a pull mechanism of about $100 million for an NTD product, these returns do not cover the investment needed for the drug or vaccine development process that may exceed $1 billion [33,47]. Additionally, although the program encourages NTD product development, it does not guarantee companies to cooperate with international organizations or country governments to ensure affordable and widespread access to these products in the US or in countries with significant NTD burdens [47,49]. Suggestions to improve the program range from providing companies with R&D grants and facilitating AMCs, adjusting the length of exclusivity, and ensuring plans to allow for the widespread access of these approved lifesaving drugs and vaccines.

### Orphan products designation

In the late 1970s, it became apparent there was a lack of medical products to treat diseases and conditions that affected a small proportion of the US population. Due to the rare occurrence of such diseases and conditions, pharmaceutical companies were not willing to invest in R&D of these products due to small sales that would not cover the costs of product development, resulting in financial losses. To address this, Congress passed the Orphan Drug Act of 1983 on January 4, 1983 to stimulate the development of drugs for such rare diseases [50]. In 1984, Congress amended the Act to define rare diseases as those affecting “less than 200,000 persons in the United States” or more than 200,000 persons, but for whom “there is no reasonable expectation that the cost of developing and making available in the United States a drug for such disease or condition will be recovered from the sale in the United States” [51,52]. Some tropical disease products may qualify for designation as orphan drugs because these products may be used to treat diseases that affect fewer than 200,000 people in the US. The Act was subsequently modified in 1985 and 1988 to extended marketing exclusivity to patentable as well as unpatentable drugs and required companies to apply for orphan drug status prior to application submission, respectively [52]. Today, the Orphan Drug Program provides companies with numerous incentives to spur the innovation and development of products for such rare diseases including: (1) tax credits for qualified clinical trials (equal to 50% of clinical investigation expenses); (2) 7-year marketing exclusivity to the first company obtaining FDA approval of an orphan designated drug; (3) exemption/waiver of PDUFA application filing fees; (4) assistance in the drug development process; and (5) eligibility for Orphan Products Grant funding [53,54].

**Fig 3:**
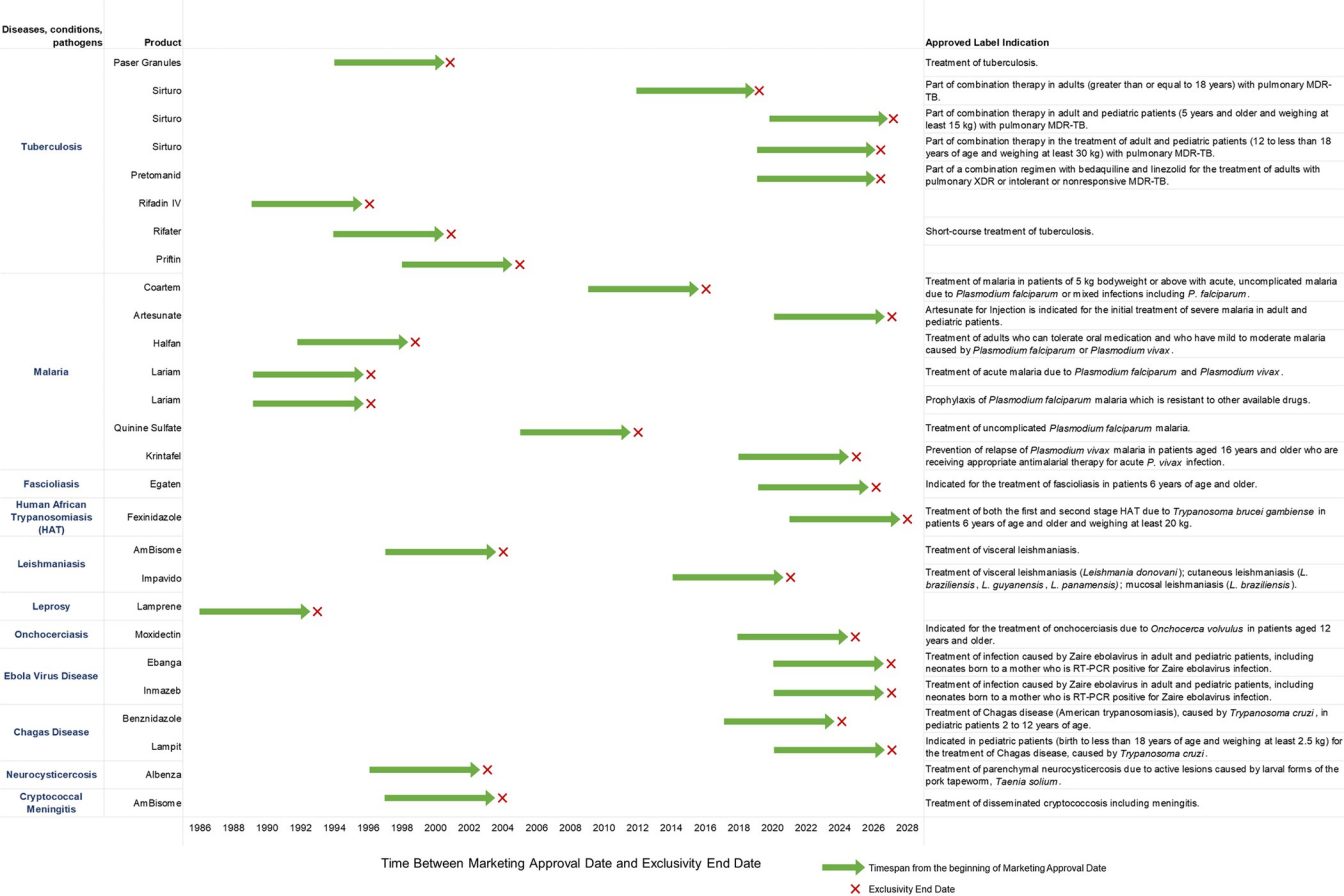
Tropical disease products approved under the Orphan Drug Designation Program by the US FDA. The figure shows products approved by tropical disease, the approved treatment indication, and the market exclusivity period after approval between January 1983 and July 2022. Data was extracted from FDA Orphan Drug Designations and Approvals Database (https://www.accessdata.fda.gov/scripts/opdlisting/oopd/index.cfm). FDA, Food and Drug Administration; HAT, Human African trypanosomiasis.

The FDA Office of Orphan Products Development (OOPD) supports the development and evaluation of new products, including drugs, biologics, and medical devices, for rare diseases. Before a company files for an NDA or BLA, the company must first submit a request to OOPD for an orphan designation. Once the request is approved, the product is said to have “orphan status” [53,54]. After approval of the “orphan product” status, the NDA or BLA application is reviewed by relevant FDA centers for safety and efficacy. Overall, 27 products for 11 tropical diseases (tuberculosis, malaria, fascioliasis, human African trypanosomiasis, leishmaniasis, leprosy, onchocerciasis, Ebola virus disease, Chagas disease, neurocysticercosis, and cryptococcal meningitis) have received orphan drug status and have been approved for use by FDA (Table 2, Fig 3). Recently, in 2020, 2 orphan designated drugs were approved for the treatment of Ebola virus disease [55,56]. The full list of FDA approved orphan designated products can be found in S2 Table.

**Table 2:** Number of products and grants approved by FDA as part of the Orphan Products Designation Program and the Orphan Products Grant Program, respectively.

Disease or condition	Number of Orphan Designated Products approved by FDA	Number of Orphan Designated Grants awarded by FDA
Brucellosis	0	0
Buruli ulcer	0	0
Chagas disease	2	0
Cholera	0	0
Cryptococcal meningitis	1	0
Dengue and Chikungunya	0	0
Dracunculiasis (Guinea-worm disease)	0	0
Filovirus diseases	2	0
Foodborne trematodiases (Fascioliasis, opisthorchiasis, and paragonimiasis)	1	0
Human African trypanosomiasis (sleeping sickness)	1	0
Lassa fever	0	0
Leishmaniasis	2	0
Leprosy (Hansen’s disease)	1	0
Lymphatic filariasis	0	0
Malaria	7	3
Onchocerciasis (river blindness)	1	0
Rabies	0	0
Schistosomiasis	0	0
Soil-transmitted helminthiases	0	0
Neurocysticercosis	1	1
Trachoma	0	0
Tuberculosis	8	4
Yaws	0	0
Zika virus disease	0	0

Orphan designations for diseases recognized as “tropical diseases” in Section 524(a)(3) of the FD&C Act were extracted with relevant search terms.

FDA, Food and Drug Administration; FD&C, Federal Food, Drug, and Cosmetic.

### Orphan Products Grant Program

The Orphan Drug Act of 1983 also created the Orphan Products Grants Program wherein grants are awarded to clinical investigators to support the clinical development of products against rare diseases [57,58]. Since the program’s inception in 1983, OOPD has granted funds totaling over $420 million to over 700 studies [59]. Since 1983, of all studies funded by this program (*n* = 678), only 8 studies have been funded for the development of tropical disease products (Table 2,); of these 8 funded studies, 4 are for tuberculosis, 3 for malaria, and 1 for neurocysticercosis—only neurocysticercosis is considered an NTD by WHO. The full list of orphan products grants awarded by FDA can be found in S3 Table.

### Generating Antibiotic Incentives Now (GAIN) and qualified infectious disease product (QIDP)

**Fig 4:**
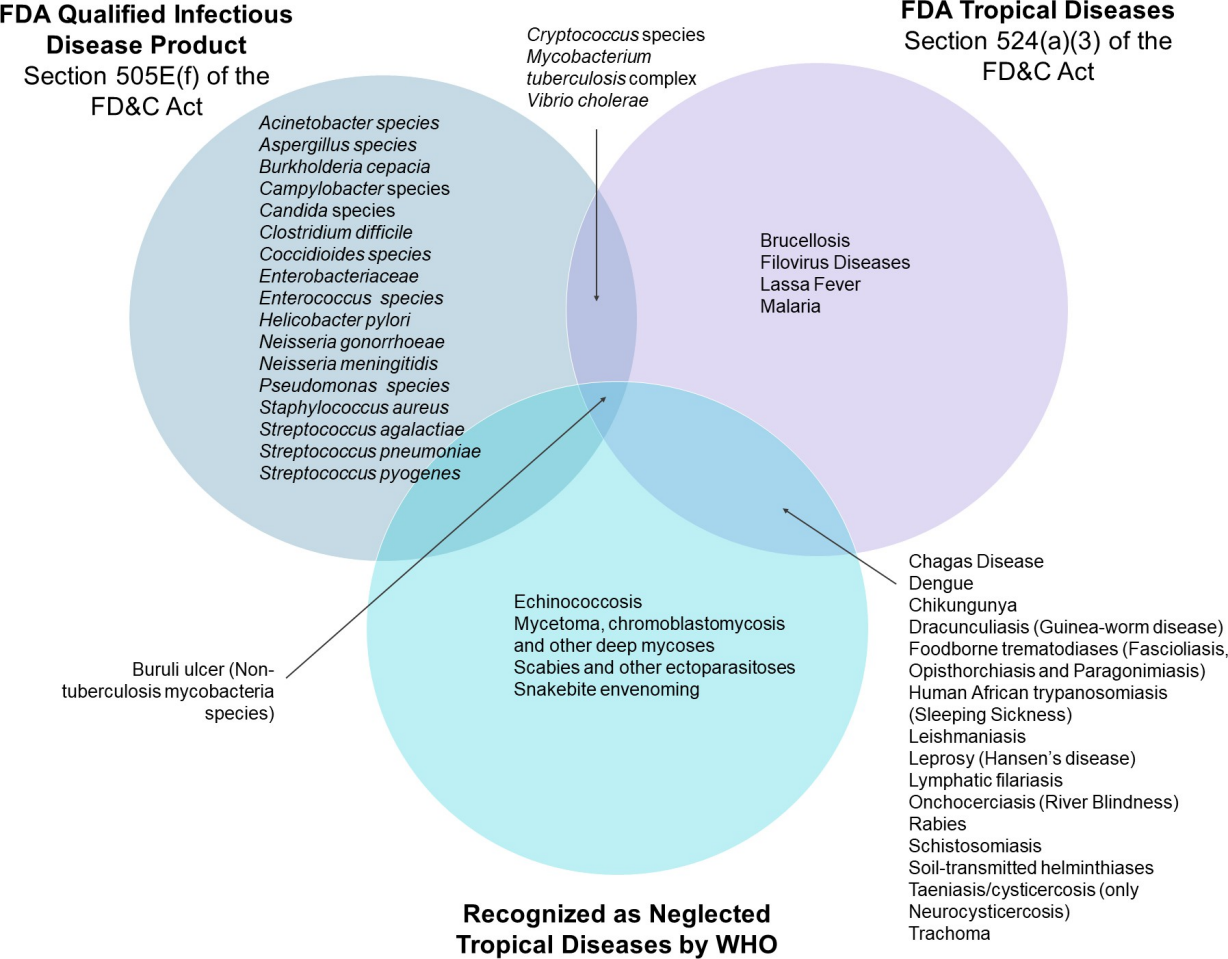
Overlap between diseases considered as QIDPs by FDA, tropical diseases by FDA, and NTDs by WHO. Information for this figure was generated using Section 505E(f) of the FD&C Act, Section 524(a)(3) of the FD&C Act and WHO’s list of NTDs. FDA, Food and Drug Administration; FD&C, Federal Food, Drug, and Cosmetic; NTD, neglected tropical disease; QIDP, qualified infectious disease product; WHO, World Health Organization.

On July 9, 2012, under Section 505E of the FD&C Act, Congress implemented the Food and Drug Administration Safety and Innovation Act (FDASIA) creating the “Generating Antibiotic Incentives Now (GAIN)” provisions [60,61]. GAIN provisions offer incentives to companies for the development of antibacterial and antifungal products that treat serious or life-threatening infections. GAIN also allows FDA to designate certain antimicrobials as qualified infectious disease products (QIDPs). QIDPs include antibacterial and antifungal drugs that can be used against antibacterial or antifungal resistant pathogens, including novel or emerging infectious pathogens and those qualifying pathogens listed by the Secretary under subsection (f) of Section 505E(f) of the FD&C Act [61,62]. Of this list of 21 qualifying pathogens [63], 4 pathogens (*Cryptococcus* species, *Mycobacterium tuberculosis* complex, non-tuberculous mycobacteria species, and *Vibrio cholerae*) are also considered as causative agents of “tropical diseases” by Section 524(a)(3) of the FD&C Act (cryptococcal meningitis, tuberculosis, Buruli ulcer, and cholera, respectively). Importantly, of these 4 pathogens, only Buruli ulcer is considered an NTD by WHO (Fig 4).

The primary incentive of GAIN is to extend the marketing exclusivity period of an approved QIDP by 5 years—during this period, these antimicrobial drugs can be sold without generic competition. This 5-year exclusivity extension is added to any exclusivity for which the application qualifies upon approval [61]. Other incentives include the ability to gain priority review and “fast-track” designations. Although the need for this legislation has been welcomed by many experts [64], studies have indicated that GAIN may not have been entirely successful in incentivizing antimicrobial development to satisfy unmet health needs [65].

### Humanitarian Use Device Designation (HUD) and Humanitarian Device Exemption (HDE)

The Humanitarian Use Device Designation (HUD) and Humanitarian Device Exemption (HDE) programs are additional programs that may have an impact on stimulating the development of NTD products to aid in disease diagnosis [66]. To create new alternate regulatory pathways for medical devices that can be used specifically for rare diseases (affecting fewer than 4,000 people in the US), Congress authorized the Safe Medical Devices Act of 1990 implementing the HDE regulatory pathway [67,68]. A subsequent amendment in 2016 defined HUDs as “a medical device intended to benefit patients in the treatment or diagnosis of a disease or condition that affects or is manifested in not more than 8,000 individuals in the United States per year” [68,69]. Once a device application receives an HUD designation from OOPD, the company can submit the HDE marketing application to respective centers for review. As part of the HDE regulatory pathway, devices are exempt from the requirements of establishing reasonable assurance of effectiveness [68].

### NTD guidance documents

FDA has finalized numerous guidance documents to help inform the product development process or regulatory policies related to tropical diseases. These documents, which are nonbinding and not enforceable, provide a glimpse into FDA’s current-thinking about regulatory policy or product development issues and are intended to guide industry as they navigate regulatory processes. The process of developing guidance document is transparent and inclusionary, with the public able to submit comments on draft guidance documents before they are finalized by FDA [70]. In July 2014, FDA published a guidance specifically for developing drugs for treatment or prevention of “neglected tropical diseases of the developing world” [71]. In addition to providing information on nonclinical and clinical development of the drug, this guidance also provides industry with regulatory tools that can be used in drug development for NTDs including orphan product designation, expedited programs, tropical disease priority review voucher, and qualified infectious disease products. A list of final guidance documents related to incentive programs and tropical disease product development is provided in S4 Table.

### Additional FDA efforts

In addition to the programs described above, FDA also provides 4 expedited programs intended to expedite and facilitate the development and review of new drugs that may address serious or life-threatening conditions—fast track designation, breakthrough therapy designation, accelerated approval, and priority review designation [72]. FDA has also collaborated with both national and international stakeholders to advance therapeutics for neglected diseases [66]. For example, FDA works with NIH on the Therapeutics for Rare and Neglected Diseases Program. FDA has also held numerous workshops on orphan product designation in the US, Europe, and India to inform and collaborate with national and international stakeholders. Additionally, FDA, through the Center for Biologics Evaluation and Research (CBER), engages with WHO’s Developing Country Vaccine Regulator Network (DCVRN), a WHO-funded network of national regulatory authorities to build regulatory capacities in respective countries.

## Discussion

Despite the increased attention towards NTDs, efforts to ensure access to lifesaving medical products falls far short of requirements needed. As shown in this review, a significant number of tropical disease products approved by FDA using these regulatory programs are indicated for tuberculosis and malaria—diseases that are not considered as NTDs by WHO. Of the 27 diseases and conditions that are eligible to receive tropical disease PRVs from FDA, no drug or vaccine has been approved and granted a PRV for 18 diseases including WHO-defined NTDs such as blinding trachoma, yaws, Buruli ulcer, dracunculiasis, schistosomiasis, soil-transmitted helminthiasis, or chikungunya virus disease (Fig 2). An assessment of the Orphan Products Program shows that 15 of the 27 approved orphan designated products for “tropical diseases” were indicated for tuberculosis and malaria; only 9 products were developed for diseases considered NTDs by WHO (fascioliasis, human African trypanosomiasis, leishmaniasis, leprosy, onchocerciasis, Chagas disease, and neurocysticercosis) (Table 2). Additionally, of all funded grants under the Orphan Products Grant Program, only 1.1% (8/678) were for tropical disease products—a majority of which were for products against tuberculosis and malaria (S3 Table). Most drugs and vaccines developed under these 3 FDA incentive programs are for diseases that have large funding investments, adding to the significant gap in funding and R&D efforts directed towards the “big three” diseases, while sidelining other neglected diseases. Indeed, an analysis of official development assistance (ODA) commitments for control of infectious disease between 2003 and 2007 reported only 0.6% of total annual health ODA directed to combat NTDs; in contrast, ODA health shares for HIV/AIDS was 36.3%, for malaria 3.6%, and for tuberculosis 2.2% [73]. Recently, the 2019 Policy Cures Research G-FINDER survey, tracking annual investment into R&D for global health, reported that 71% of total investments in neglected diseases were concentrated on HIV/AIDS, tuberculosis, and malaria; investments in R&D for several neglected diseases such as human African trypanosomiasis, lymphatic filariasis, and helminthiases declined significantly [74,75]. Furthermore, between 2000 and 2011, only 4% of all approved new products and only 1% of all approved new chemical entities (NCEs) were indicated for neglected diseases [27]. While the increased and accelerated efforts towards combating the “big three” diseases—HIV/AIDS, malaria, and tuberculosis—have been long overdue and necessary due to their own “neglected” past and burden on populations globally, it is crucial for governments, policymakers, and other stakeholders to address the significant lack of resources and funding for the other sidelined neglected diseases.

The 2030 NTD Roadmap also highlights the lack of diagnostic tools for NTDs as a significant hurdle to the goals of NTD control, elimination, and eradication [26]. For many NTDs such as foodborne trematodiases, leishmaniases, and echinococcoses, no diagnostic tests are available either for the screening or surveillance stages. For multiple NTDs such as Chagas disease, leishmaniasis, and Buruli ulcer, while diagnostic tests confirming diagnosis exist, major modifications are required to these tests to meet the 2030 NTD Roadmap goals. While an assessment of the NTD diagnostic tests approved by FDA under appropriate regulatory pathways is beyond the scope of this review, it is crucial for national regulatory agencies to work with companies to ensure the innovation and availability of NTD diagnostic tools. Additionally, the emergence of antimicrobial resistance (AMR) in NTDs is another key reason why continued investment in NTD R&D is required for the development of drugs, vaccines, and diagnostics. Since, the majority of NTD programs rely on the use of medicines as part of control, elimination, and eradication efforts, the emergence of resistance to these medicines threatens advances and progress made by NTD programs. This requires the availability of diagnostic tools to detect the emergence of AMR and the development and availability of second-line medicines that can be used in NTD programs.

While incentive programs to spur innovation of NTDs have been welcomed as a step towards improving R&D for NTD products, crucial R&D gaps persist as shown in this review. Additional efforts are required to develop products specifically for NTDs as a vast majority of products developed through programs such as the tropical disease PRV program and the Orphan Product Designation program have been for tuberculosis and malaria. The Orphan Product Designation program has approved only 9 products for diseases considered as NTD by WHO, while the tropical disease PRV program has only approved 5 products for 4 diseases considered NTDs by WHO. This suggests that although these programs have an impact on company R&D efforts, the lack of potential markets continues to be a major hindrance to NTD R&D as suggested by previous studies [45].

Efforts to improve product R&D specifically for overlooked NTDs could include the use of AMCs wherein donor organizations, including governments or foundations, pledge to provide a viable market for an NTD medical product once it is successfully developed. AMCs have been successfully implemented during the ongoing Coronavirus Disease 2019 (COVID-19) pandemic, wherein many countries and international organizations signed advance purchase agreements with companies developing COVID-19 vaccines, resulting in availability of vaccine supplies [76,77]. AMCs could help reduce reliance on philanthropy and drug donations by pharmaceutical companies, which is prevalent in the NTD space, and help in the development of a business model that is likely to be sustainable and result in continued innovation of NTD products. In addition to AMCs, leveraging on public–private partnerships for NTD product development is another possible solution to address the lack of investment in NTD R&D. Such partnerships could take the form of developing collaborative frameworks for prioritization of candidate drugs, vaccines, and diagnostics; operation of clinical trials; and coordinating regulatory processes. Lastly, to maintain commitment towards investing in NTD R&D, regular participation in multi-stakeholder consortia to revisit and reassess unmet needs is critical. Indeed, by adopting a model wherein endorsers of the London Declaration, including academic and research institutions, government, multilaterals, donors, and pharmaceutical companies, convene at regular intervals, progress towards the overall goal of combating NTDs can be monitored and assessed.

A comprehensive policy toolkit that incorporates prioritizing and coordinating NTD research, the availability of R&D funding for NTDs, the enhancement of regulatory mechanisms to facilitate NTD product innovation, and mechanisms to ensure provision of and access to these products is essential. With more than 1 billion people in the world suffering from at least 1 NTD, it is vital to “end the neglect” of these historically neglected diseases.

**Key learning points**

1. Numerous FDA regulatory pathways and programs have been authorized by the US government to aid in the development of drugs, vaccines, and devices for combatting NTDs.
2. While these programs are deemed important by regulatory experts, policy experts, pharmaceutical company executives, and other stakeholders, a clear picture on the impact of many of these pathways and programs on NTD product R&D is lacking.
3. Majority of products approved by FDA using these programs and pathways are for diseases such as tuberculosis and malaria, which are not considered as NTDs by the World Health Organization. No product for NTDs such as blinding trachoma, yaws, Buruli ulcer, dracunculiasis, schistosomiasis, soil-transmitted helminthiasis, or chikungunya virus disease have been approved using the “priority review vouchers” and “orphan product status” programs.
4. Dedicated efforts to direct resources and attention to NTDs by the government is required; these efforts can include use of a combination of incentive programs and leveraging public private partnerships for NTD R&D.

**Five key papers in the field**

1. Pedrique B, Strub-Wourgaft N, Some C, Olliaro P, Trouiller P, Ford N, et al. The drug and vaccine landscape for neglected diseases (2000–11): a systematic assessment. Lancet Glob Health. 2013;1(6):e371-e379. doi:10.1016/S2214-109X(13)70078-0
2. Ridley DB, Grabowski HG, Moe JL. Developing Drugs for Developing Countries. Health Aff. 2006;25(2):313–324. doi:10.1377/hlthaff.25.2.313
3. Robertson AS, Stefanakis R, Joseph D, Moree M. The Impact of the US Priority Review Voucher on Private-Sector Investment in Global Health Research and Development. PLoS Negl Trop Dis. 2012;6(8):e1750. doi:10.1371/journal.pntd.0001750
4. Miller KL, Mueller C, Liu G, Miller Needleman KI, Maynard J. FDA orphan products clinical trial grants: assessment of outcomes and impact on rare disease product development. Orphanet J Rare Dis. 2020;15(1):234. doi:10.1186/s13023-020-01514-5
5. Darrow JJ, Kesselheim AS. Incentivizing Antibiotic Development: Why Isn’t the Generating Antibiotic Incentives Now (GAIN) Act Working? Open Forum Infect Dis. 2020;7(1):ofaa001. doi:10.1093/ofid/ofaa001

## Supporting information

S1 Table Drug and vaccine products approved by FDA under the tropical disease PRV program. The list includes date of product approval, company sponsor and PRV recipient, and links to FDA approval packages and letters.
(DOCX)

S2 Table Tropical disease products designated as orphan products and approved by FDA under the Orphan Drug Designation Program. The list includes date of orphan designation status, orphan designation, marketing approval date, exclusivity end date, and name of sponsor company. The FDA orphan drug database was accessed through https://www.accessdata.fda.gov/scripts/opdlisting/oopd/. Search criteria were “only approved products” from January 1, 1983 until July 6, 2022 (*N* = 1,070) and the output format was an excel file. Orphan designations for diseases recognized as “tropical diseases” in Section 524(a)(3) of the FD&C Act were extracted with relevant search terms (*n* = 27).
(DOCX)

S3 Table List of grants awarded by FDA through the Orphan Products Grants Program to support the development of safe and effective tropical disease medical products. The list includes the product name, indication, start date, end date, title of study, and the company. The FDA orphan products grants program database was accessed through https://www.accessdata.fda.gov/scripts/opdlisting/oopdgrants/. Search criteria were “all funded grants (current and previous)” from January 1, 1983 until July 22, 2022 (*N* = 678) and the output format was an excel file. Grants for diseases recognized as “tropical diseases” in Section 524(a)(3) of the FD&C Act were extracted with relevant search terms (*n* = 8).
(DOCX)

S4 Table List of guidance documents published by FDA related to production of medical products for tropical diseases. This list includes guidance documents that have been finalized as of July 2022. Guidance documents were extracted from https://www.fda.gov/regulatory-information/search-fda-guidance-documents using relevant search terms.
(DOCX)
