# Supplementary material for: The United States Food and Drug Administration (FDA) regulatory response to combat neglected tropical diseases (NTDs): A review
Source: PLoS Negl Trop Dis. 2023 Jan 12;17(1):e0011010. doi: 10.1371/journal.pntd.0011010 (PMC9836280; doi:10.1371/journal.pntd.0011010)
Supplement: S1 Table — The list includes date of product approval, company sponsor and PRV recipient, and links to FDA approval packages and letters. (DOCX) [file pntd.0011010.s001.docx]

**S1 Table. Drug and vaccine products approved by FDA under the Tropical Disease PRV Program.** The list includes date of product approval, company sponsor & PRV recipient and links to FDA approval packages and letters

| **Product** | **Disease, Condition, Pathogens** | **Date of Product Approval** | **Company Sponsor and PRV Recipient** | **FDA Approval Package/Letter URL** |
| --- | --- | --- | --- | --- |
| Coartem (artemether/lumefantrine) | Malaria | 04/07/2009 | Novartis | <https://www.accessdata.fda.gov/drugsatfda_docs/nda/2009/022268s000_SumR.pdf> |
| Sirturo (bedaquiline) | Tuberculosis | 12/28/2012 | Janssen Research and Development (JNJ) | <https://www.accessdata.fda.gov/drugsatfda_docs/appletter/2012/204384Orig1s000ltr.pdf> |
| Impavido (miltefosine) | Leishmaniasis | 03/19/2014 | Paladin Therapeutics, Inc | <https://www.accessdata.fda.gov/drugsatfda_docs/appletter/2014/204684Orig1s000ltr.pdf> |
| Vaxchora | Cholera | 06/10/2016 | PaxVax Bermuda Ltd | <https://www.fda.gov/news-events/press-announcements/fda-approves-vaccine-prevent-cholera-travelers> |
| Benznidazole | Chagas disease (American trypanosomiasis) | 08/29/2017 | Chemo Research, S.L | <https://www.accessdata.fda.gov/drugsatfda_docs/appletter/2017/209570Orig1s000ltr.pdf> |
| Moxidectin | Onchocerciasis | 06/13/2018 | Medicines Development Limited (trading as Medicines Development for Global Health) | <https://www.accessdata.fda.gov/drugsatfda_docs/appletter/2018/210867Orig1s000Ltr.pdf> |
| Krintafel (tafenoquine) | Malaria | 07/20/2018 | GSK | <https://www.accessdata.fda.gov/drugsatfda_docs/nda/2018/210795Orig1s000Approv.pdf> |
| Egaten (triclabendazole) | Fascioliasis | 02/13/2019 | Novartis Pharmaceuticals Corporation | <https://www.accessdata.fda.gov/drugsatfda_docs/appletter/2019/208711Orig1s000ltr.pdf> |
| Dengvaxia | Dengue | 05/01/2019 | Sanofi Pasteur Inc | <https://www.fda.gov/media/124402/download> |
| Pretomanid | Tuberculosis | 08/14/2019 | The Global Alliance for TB Drug Development, Inc. (TB Alliance) | <https://www.accessdata.fda.gov/drugsatfda_docs/nda/2019/212862Orig1s000Approv.pdf> |
| Ervebo | Ebola Zaire (Filovirus) | 12/19/2019 | Merck Sharp & Dohme Corp | <https://www.fda.gov/media/133757/download> |
| Lampit (nifurtimox) | Chagas disease | 08/06/2020 | Bayer HealthCare Pharmaceuticals, Inc. | <https://www.accessdata.fda.gov/drugsatfda_docs/appletter/2020/213464Orig1s000ltr.pdf> |
