# Supplementary material for: The United States Food and Drug Administration (FDA) regulatory response to combat neglected tropical diseases (NTDs): A review
Source: PLoS Negl Trop Dis. 2023 Jan 12;17(1):e0011010. doi: 10.1371/journal.pntd.0011010 (PMC9836280; doi:10.1371/journal.pntd.0011010)
Supplement: S2 Table — The list includes date of orphan designation status, orphan designation, marketing approval date, exclusivity end date, and name of sponsor company. The FDA orphan drug database was accessed through https://www.accessdata.fda.gov/scripts/opdlisting/oopd/. Search criteria were “only approved products” from January 1, 1983 until July 6, 2022 (N = 1,070) and the output format was an excel file. Orphan designations for diseases recognized as “tropical diseases” in Section 524(a)(3) of the FD&C Act were extracted with relevant search terms (n = 27). (DOCX) [file pntd.0011010.s002.docx]

**S2 Table. Tropical disease products designated as orphan products and approved by FDA under the** **Orphan Drug Designation Program.** The list includes date of orphan designation status, orphan designation, marketing approval date, exclusivity end date and name of sponsor company. The FDA orphan drug database was accessed through <https://www.accessdata.fda.gov/scripts/opdlisting/oopd/>. Search criteria were “only approved products” from January 1, 1983 until July 6, 2022 (N = 1070) and the output format was an excel file. Orphan designations for diseases recognized as ‘tropical diseases’ in Section 524(a)(3) of the FD&C Act were extracted with relevant search terms (n = 27).

| **Product** | **Sponsor Company** | **Disease, Condition, Pathogens** | **Date Designated** | **Orphan Designation** | **Marketing Approval Date** | **Exclusivity End Date** |
| --- | --- | --- | --- | --- | --- | --- |
| Paser Granules (Aminosalicylic acid) | Jacobus Pharmaceutical Company | Tuberculosis | 2/19/1992 | Treatment of tuberculosis infections | 6/30/1994 | 6/30/2001 |
| Sirturo (bedaquiline) | Janssen Research & Development, LLC | Tuberculosis | 1/10/2005 | Treatment of active tuberculosis | 12/28/2012 | 12/28/2019 |
| Sirturo (bedaquiline) | Janssen Research & Development, LLC | Tuberculosis | 1/10/2005 | Treatment of active tuberculosis | 5/27/2020 | 5/27/2027 |
| Sirturo (bedaquiline) | Janssen Research & Development, LLC | Tuberculosis | 1/10/2005 | Treatment of active tuberculosis | 8/9/2019 | 8/9/2026 |
| Pretomanid | Global Alliance for TB Drug Development | Tuberculosis | 7/5/2007 | Treatment of tuberculosis | 8/14/2019 | 8/14/2026 |
| Rifadin IV (Rifampin) | Hoechst Marion Roussel | Tuberculosis | 12/9/1985 | For antituberculosis treatment where use of the oral form of the drug is not feasible. | 5/25/1989 | 5/25/1996 |
| Rifater (Rifampin, isoniazid, pyrazinamide) | Hoechst Marion Roussel | Tuberculosis | 9/12/1985 | For the short-course treatment of tuberculosis. | 5/31/1994 | 5/31/2001 |
| Priftin (Rifapentine) | Hoechst Marion Roussel | Tuberculosis | 6/9/1995 | Treatment of pulmonary tuberculosis. | 6/22/1998 | 6/22/2005 |
| Coartem (artemether/ lumefantrine) | Novartis Pharmaceuticals | Malaria | 8/31/2007 | For the treatment of infections due to *Plasmodium falciparum* or mixed infections including *P. falciparum.* | 4/7/2009 | 4/7/2016 |
| Artesunate | Amivas Inc. | Malaria | 3/28/2006 | Immediate treatment of malaria | 5/26/2020 | 5/26/2027 |
| Halfan (Halofantrine) | SmithKline Beecham Pharmaceuticals | Malaria | 11/4/1991 | Treatment of mild to moderate acute malaria caused by susceptible strains of *P. falciparum* and *P. vivax.* | 7/24/1992 | 7/24/1999 |
| Lariam (mefloquine HCl) | Hoffmann-La Roche, Inc. | Malaria | 4/13/1988 | For use in the treatment of acute malaria due to *Plasmodium falciparum* and *Plasmodium viva*x, and for the prophylaxis of *Plasmodium falciparum* malaria which is resistant to other available drugs | 5/2/1989 | 5/2/1996 |
| Lariam (mefloquine HCl) | Hoffmann-La Roche, Inc. | Malaria | 4/13/1988 | For use in the treatment of acute malaria due to *Plasmodium falciparum* and *Plasmodium vivax*, and for the prophylaxis of *Plasmodium falciparum* malaria which is resistant to other available drugs | 5/3/1989 | 5/3/1996 |
| Quinine Sulfate | AR Holding Company, Inc. | Malaria | 6/3/2004 | Treatment of malaria | 8/12/2005 | 8/12/2012 |
| Krintafel (tafenoquine) | GlaxoSmithKline Intellectual Property Development Ltd. England | Malaria | 1/15/2013 | Treatment of malaria | 7/20/2018 | 7/20/2025 |
| Egaten (triclabendazole) | Novartis Pharmaceuticals Corporation | Fascioliasis | 4/17/2017 | Treatment of fascioliasis | 2/13/2019 | 2/13/2026 |
| Fexinidazole | Sanofi US Services Inc., A SANOFI COMPANY | Human African trypanosomiasis | 4/4/2016 | Treatment of human African trypanosomiasis (HAT) or sleeping sickness | 7/16/2021 | 7/16/2028 |
| AmBisome (Liposomal amphotericin B) | Fujisawa USA, Inc. | Leishmaniasis | 12/6/1996 | Treatment of visceral leishmaniasis. | 8/11/1997 | 8/11/2004 |
| Impavido (miltefosine) | Knight Therapeutics (USA) | Leishmaniasis | 10/10/2006 | Treatment of leishmaniasis. | 3/19/2014 | 3/19/2021 |
| Lamprene (Clofazimine) | Novartis Pharmaceutical Corporation | Leprosy | 6/11/1984 | Treatment of lepromatous leprosy, including dapsone-resistant lepromatous leprosy and lepromatous leprosy complicated by erythema nodosum leprosum. | 12/15/1986 | 12/15/1993 |
| Moxidectin | Medicines Development Limited | Onchocerciasis | 9/29/2010 | Treatment of onchocerciasis volvulus in children and adults | 6/13/2018 | 6/13/2025 |
| Ebang (ansuvimab-zykl) | Ridgeback Biotherapeutics, LP | Ebola | 5/8/2019 | Treatment of Ebola Virus Disease | 12/21/2020 | 12/21/2027 |
| Inmazeb (atoltivimab, maftivimab, and odesivimab-ebgn) | Regeneron Pharmaceuticals, Inc. | Ebola | 7/14/2016 | Treatment of Ebola virus infection | 10/14/2020 | 10/14/2027 |
| Benznidazole | Chemo Research, S.L. | Chagas Disease | 4/14/2014 | Treatment of Chagas disease | 8/29/2017 | 8/29/2024 |
| Lampit (nifurtimox) | Bayer HealthCare Pharmaceuticals, Inc. | Chagas Disease | 8/5/2010 | Treatment of Chagas disease (American Trypanosomiasis) caused by *T. cruzi* | 8/6/2020 | 8/6/2027 |
| Albenza (Albendazole) | Impax Laboratories, LLC | Neurocysticercosis | 1/18/1996 | Treatment of neurocysticercosis due to *Taenia solium* as: 1) chemotherapy of parenchymal, subarachnoidal and racemose (cysts in spinal fluid) neurocysticercosis in symptomatic cases and 2) prophylaxis of epilepsy and other sequelae in asymptomatic neurocys | 6/11/1996 | 6/11/2003 |
| AmBisome (Liposomal amphotericin B) | Fujisawa USA, Inc. | Cryptococcal Meningitis | 12/10/1996 | Treatment of cryptococcal meningitis. | 8/11/1997 | 8/11/2004 |
