# Supplementary material for: The United States Food and Drug Administration (FDA) regulatory response to combat neglected tropical diseases (NTDs): A review
Source: PLoS Negl Trop Dis. 2023 Jan 12;17(1):e0011010. doi: 10.1371/journal.pntd.0011010 (PMC9836280; doi:10.1371/journal.pntd.0011010)
Supplement: S3 Table — The list includes the product name, indication, start date, end date, title of study, and the company. The FDA orphan products grants program database was accessed through https://www.accessdata.fda.gov/scripts/opdlisting/oopdgrants/. Search criteria were “all funded grants (current and previous)” from January 1, 1983 until July 22, 2022 (N = 678) and the output format was an excel file. Grants for diseases recognized as “tropical diseases” in Section 524(a)(3) of the FD&C Act were extracted with relevant search terms (n = 8). (DOCX) [file pntd.0011010.s003.docx]

**S3 Table. List of grants awarded by FDA through the** **Orphan Products Grants Program to support the development of safe and effective tropical disease medical products.** The list includes the product name, indication, start date, end date, title of study and the company. The FDA orphan products grants program database was accessed through <https://www.accessdata.fda.gov/scripts/opdlisting/oopdgrants/>. Search criteria were “all funded grants (current and previous)” from January 1, 1983 until July 22, 2022 (N = 678) and the output format was an excel file. Grants for diseases recognized as ‘tropical diseases’ in Section 524(a)(3) of the FD&C Act were extracted with relevant search terms (n = 8).

| **Product Name** | **Indication** | **Start date** | **End date** | **Title** | **Company** |
| --- | --- | --- | --- | --- | --- |
| PA-824 (Pretomanid) | Pulmonary Tuberculosis | 8/1/2014 | 7/31/2022 | Phase 2 Study of PA-824 for the Treatment of Pulmonary Tuberculosis | Johns Hopkins University |
| Rifapentine | Pulmonary Tuberculosis | 1/1/2009 | 8/31/2014 | Phase II Study of Daily Rifapentine for Pulmonary Tuberculosis | Johns Hopkins University |
| Rifampin (Rifadin), Merrem (meropenem) & Augmentin (Amoxicillin/clavulanate) | Pulmonary Tuberculosis | 8/1/2017 | 6/30/2022 | Phase 2a Study of Rifampin, Merrem & Augmentin for the Treatment of Pulmonary Tuberculosis | Johns Hopkins University |
| Aminosidine | Tuberculosis | 8/1/1995 | 1/31/1999 | A Pilot Study to Determine the PK and EBA of Aminosidine | University of Illinois at Chicago |
| AQ-13 | Drug Resistant Malaria | 3/9/2009 | 3/8/2018 | Phase 2 Studies of AQ-13 for the Treatment of Drug Resistant Malaria | Tulane University |
| Desferrioxamine | Cerebral malaria | 9/1/1991 | 8/31/1997 | Iron Chelation with Desferrioxamine for Cerebral Malaria | Pennsylvania State University |
| Investigational aminoquinoline | Treatment of *Plasmodium falciparum* infection malaria | 9/1/1999 | 1/31/2003 | Phase I Studies of an Investigational Aminoquinoline against *Plasmodium falciparum* Infection | Tulane School of Public Health & Tropical Medicine |
| Albendazole | Epilepsy caused by neurocysticercosis | 9/30/1995 | 3/31/2002 | Effect of Albendazole Therapy on Epilepsy to Cysticercosis | Johns Hopkins University |
