# Supplementary material for: The United States Food and Drug Administration (FDA) regulatory response to combat neglected tropical diseases (NTDs): A review
Source: PLoS Negl Trop Dis. 2023 Jan 12;17(1):e0011010. doi: 10.1371/journal.pntd.0011010 (PMC9836280; doi:10.1371/journal.pntd.0011010)
Supplement: S4 Table — This list includes guidance documents that have been finalized as of July 2022. Guidance documents were extracted from https://www.fda.gov/regulatory-information/search-fda-guidance-documents using relevant search terms. (DOCX) [file pntd.0011010.s004.docx]

**S4 Table. List of guidance documents published by FDA related to production of medical products for tropical diseases.** This list includes guidance documents that have been finalized as of July 2022. Guidance documents were extracted from <https://www.fda.gov/regulatory-information/search-fda-guidance-documents> using relevant search terms.

| **Guidance Document Title** | **FDA Issuing Center** | **Date of Publication of Final Guidance** | **Link to Document** |
| --- | --- | --- | --- |
| General Principles for the Development of Vaccines to Protect Against Global Infectious Diseases: Guidance for Industry | Center for Biologics Evaluation and Research | 12/01/2011 | <https://www.fda.gov/regulatory-information/search-fda-guidance-documents/general-principles-development-vaccines-protect-against-global-infectious-diseases> |
| Expedited Programs for Serious Conditions––Drugs and Biologics | Center for Drug Evaluation and Research | 05/30/2014 | <https://www.fda.gov/regulatory-information/search-fda-guidance-documents/expedited-programs-serious-conditions-drugs-and-biologics> |
| Neglected Tropical Diseases of the Developing World: Developing Drugs for Treatment or Prevention: Guidance for Industry | Center for Drug Evaluation and Research | 07/07/2014 | <https://www.fda.gov/regulatory-information/search-fda-guidance-documents/neglected-tropical-diseases-developing-world-developing-drugs-treatment-or-prevention> |
| Meetings with the Office of Orphan Products Development: Guidance for Industry, Researchers, Patient Groups, and Food and Drug Administration Staff | Office of the Commissioner, Office of Clinical Policy and Programs, Office of Orphan Products Development | 07/09/2015 | <https://www.fda.gov/regulatory-information/search-fda-guidance-documents/meetings-office-orphan-products-development> |
| Tropical Disease Priority Review Vouchers: Guidance for Industry | Center for Drug Evaluation and Research  Center for Biologics Evaluation and Research | 10/06/2016 | <https://www.fda.gov/regulatory-information/search-fda-guidance-documents/tropical-disease-priority-review-vouchers> |
| Breakthrough Devices Program: Guidance for Industry and Food and Drug Administration Staff | Center for Devices and Radiological Health  Center for Biologics Evaluation and Research | 12/18/2018 | <https://www.fda.gov/regulatory-information/search-fda-guidance-documents/breakthrough-devices-program> |
| Humanitarian Use Device (HUD) Designations:  Guidance for Industry and Food and Drug Administration Staff | Center for Biologics Evaluation and Research  Center for Devices and Radiological Health | 09/05/2019 | <https://www.fda.gov/regulatory-information/search-fda-guidance-documents/humanitarian-use-device-hud-designations> |
| Humanitarian Device Exemption (HDE) Program: Guidance for Industry and Food and Drug Administration Staff | Center for Devices and Radiological Health  Center for Biologics Evaluation and Research | 09/06/2019 | <https://www.fda.gov/regulatory-information/search-fda-guidance-documents/humanitarian-device-exemption-hde-program> |
| Qualified Infectious Disease Product Designation Questions and Answers: Guidance for Industry | Center for Drug Evaluation and Research | 05/11/2021 | <https://www.fda.gov/regulatory-information/search-fda-guidance-documents/qualified-infectious-disease-product-designation-questions-and-answers> |
